# A Framework for Exploring Functional Variability in Olfactory Receptor Genes

**DOI:** 10.1371/journal.pone.0000682

**Published:** 2007-08-01

**Authors:** Orna Man, David C. Willhite, Chiquito J. Crasto, Gordon M. Shepherd, Yoav Gilad

**Affiliations:** 1 Department of Molecular Genetics, Weizmann Institute of Science, Rehovot, Israel; 2 Department of Structural Biology, Weizmann Institute of Science, Rehovot, Israel; 3 Department of Neurobiology, Yale University School of Medicine, New Haven, Connecticut, United States of America; 4 Department of Human Genetics, University of Chicago, Chicago, Illinois, United States of America; Purdue University, United States of America

## Abstract

**Background:**

Olfactory receptors (ORs) are the largest gene family in mammalian genomes. Since nearly all OR genes are orphan receptors, inference of functional similarity or differences between odorant receptors typically relies on sequence comparisons. Based on the alignment of entire coding region sequence, OR genes are classified into families and subfamilies, a classification that is believed to be a proxy for OR gene functional variability. However, the assumption that overall protein sequence diversity is a good proxy for functional properties is untested.

**Methodology:**

Here, we propose an alternative sequence-based approach to infer the similarities and differences in OR binding capacity. Our approach is based on similarities and differences in the predicted binding pockets of OR genes, rather than on the entire OR coding region.

**Conclusions:**

Interestingly, our approach yields markedly different results compared to the analysis based on the entire OR coding-regions. While neither approach can be tested at this time, the discrepancy between the two calls into question the assumption that the current classification reliably reflects OR gene functional variability.

## Introduction

Olfactory receptor (OR) genes, the largest gene family in mammalian genomes, constitute the basis for the sense of smell [1]–[3]. Based on their full length protein sequence similarity, mammalian OR genes are divided into two classes, 17 families and ∼250 subfamilies [4]. OR genes from the same subfamily are defined as sharing 60% or more of their overall amino acid sequence. Class I genes are closely related to OR genes found in fish and are hence referred to as ‘fish like’ [5], while class II OR genes are specific to terrestrials [3]; but see [6].

Recently, the complete OR gene repertoires of a number of mammalian species became available, permitting interspecies comparisons of complete OR gene repertoires [3], [7]–[10]. Such inter-species comparisons may increase our understanding of the similarities and differences between the sense of smell of different species. Humans, for example, have roughly 900 OR genes [3], [11], [12], but 54% of them carry one or more coding region disruptions and therefore are annotated as pseudogenes. In contrast, the mouse OR gene repertoire is ∼30% larger than that of man [7], [13], [14], but contains only 20% pseudogenes. Thus, the mouse putative functional OR gene repertoire is more than three times larger than that of humans [15]. Similarly low proportions of OR pseudogenes were found in dogs and rats [8], [9], [16]. In fact, it appears that humans have been accumulating OR pseudogenes faster than other primates [17]–[19], and as a result, have fewer intact (and putatively functional) OR genes, even when compared with chimpanzee, our closest living evolutionary relative [10].

However, nearly all mammalian OR genes are orphan receptors, as very few ligand (odorant) – receptor interactions have been demonstrated for OR genes [20]. Indeed, direct inter-species functional studies are extremely demanding and hence rare [21]. As a proxy for functional variability, similarities and differences between the protein sequence of OR genes are often used [7], [14]. In this approach, it is assumed that when orthologous OR genes are identical in sequence, both species maintain the same olfactory capability. More difficult is the interpretation of sequence *differences* between orthologous genes. To date, OR genes have been thought to have a similar function so long as they were classified into the same subfamily based on the full length of the OR protein [4], [7], [14]. This assumption remains untested.

Differences in specific binding properties are expected to be affected primarily by changes in the receptor's binding site(s) [22]–[24]. Hence, sequence similarity in binding sites, rather than in the entire protein, may be a more reliable predictor of functional similarity. To examine this possibility, we assess functional variability across OR gene repertoires of two species, mouse and human, by considering only the putative OR protein binding site [25]. We assume that OR genes with identical binding sites have the same binding properties and use Grantham's amino acid property scales [26] to model functional differences among binding sites. We note that the assumption underlying our approach is also not tested. Moreover, at this time we cannot assess whether our analysis provides more accurate functional inference than the commonly used analysis based on the entire OR coding region. Instead, we propose our approach as a second, sensible solution to a problem that to date has only been addressed using only a single type of analysis. Interestingly, we find that the two approaches yield markedly different results, with potentially important implications for the interpretation of OR gene families.

## Results

### Representation of the odorant space

The species' odorant space can be defined as the collection of all odorants that a species is capable of detecting as well as their detection thresholds. Direct mapping of a species' odorant space could be achieved by either *in vivo* testing of the detection threshold for all possible odorants, or by identifying the binding affinity properties of all functional OR genes *in vitro*. Currently, high throughput data of this type do not exist.

Instead, we take an indirect route to infer the odorant space of human and mouse by mapping the chemical distances between the putative specificity determining residues (SDRs) [25] of all OR genes. These residues were predicted based on protein sequence analysis and are supported by experimental results (see methods). In particular, a functional role for most of these putative SDRs was confirmed by a recent OR protein functional study (Kristin Schmiedeberg, Elena Shirokova, Hans-Peter Weber, Jens Reden, Thomas Hummel, Boris Schilling, Wolfgang Meyerhof, and Dietmar Krautwurst; personal communication). Henceforth, we refer to the SDR residues as the putative binding site.

For our inter-species comparison of OR repertoire variability, we use the entire human and mouse OR intact gene repertoires, the only two species for which we have well characterized repertoires from finished genomic sequence (the dog OR repertoire is not yet fully described [8] and the draft of the chimpanzee genome has relatively low coverage for this type of analysis [10]). We use previously published multiple alignment of OR genes from human and mouse [25] in order to identify the putative binding sites of all OR proteins. To estimate distances between binding sites, we construct a distance matrix for the 22 amino acid residues of each binding site, utilizing Grantham indices [26]. We visualize this distance matrix in two and/or three dimensional space by using the isomap algorithm [27], [28] for geodesic mapping (see methods). This approach assumes that proximity in binding sites reflects similarity in binding properties (i.e., that the ORs bind related odorants with similar binding affinity). Conversely, distant binding sites are assumed to have highly distinct binding properties. This set of assumptions has also been made when inferring functional variability from phylogenetic tree distances between OR genes based on the entire protein sequence (e.g., [14]).

### The human and mouse odorant spaces

**Figure 1:**
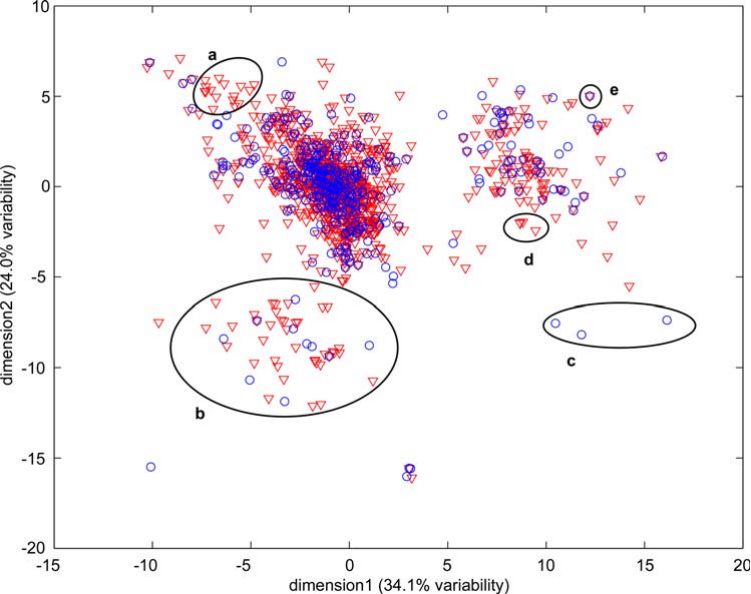
2D representation of the human (red) and mouse (blue) odorant spaces. Map regions with an over-representation of binding sites from either human or mouse are circled (a–d). An example of a pair of human-mouse orthologs (MOR27-1 and OR52P1) with identical binding site is indicated in (e).

We superimpose two-dimensional representations of the human and mouse odorant spaces to compare the relative space coverage. As can be seen in figure 1, the odorant spaces of human and mouse largely overlap. However, even at low resolution (a high resolution 3D map of the entire data is available as supplementary material at http://senselab.med.yale.edu/senselab/ORDB/ordb_ent.html), regions of the map enriched in ORs from only one of the species are readily apparent (*e.g*. regions a–d in Figure 1). We note that, as expected, representing the human and mouse OR repertoires using randomly selected groups of 22 residues of the OR protein yields vastly different results than those shown here (Figure S1).

**Figure 2:**
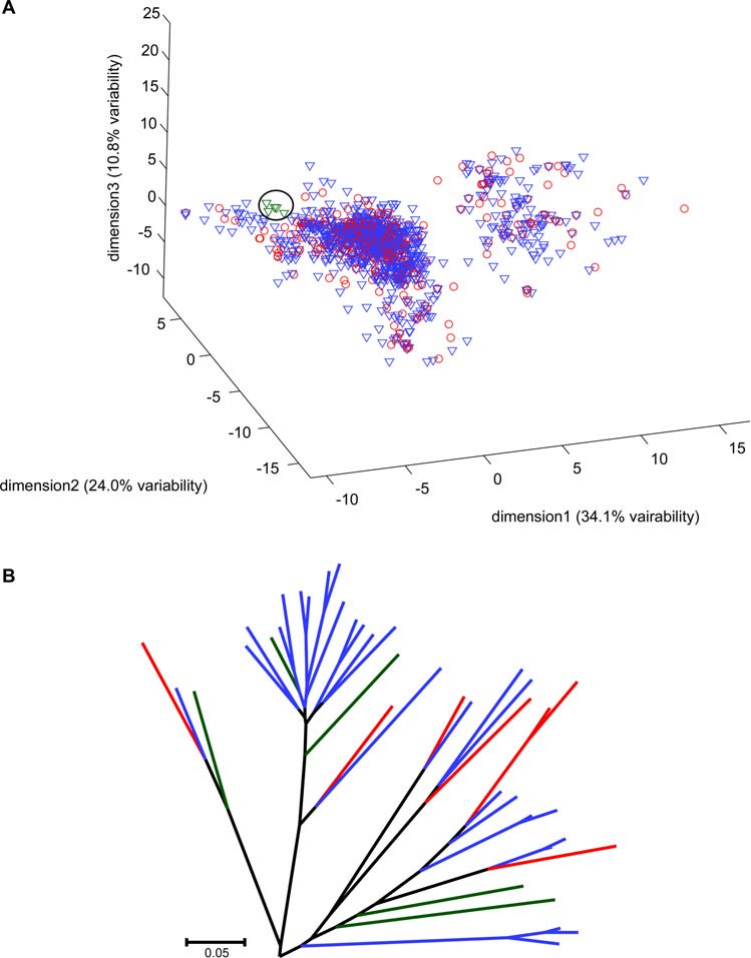
A. 3D representation of the human (red) and mouse (blue) odorant spaces. Shown in dark green is a mouse-specific cluster (consisting of MOR232-2, MOR232-5, MOR233-1, MOR233-7, MOR235-1). B. A phylogenetic tree based on the full protein sequence that includes all five genes (dark green) in the mouse specific cluster shown in A. The five genes do not form a monophyletic clade, but instead are interspersed among human (red) and other mouse (blue) genes.

To identify groups of OR genes with highly similar binding sites, we perform a clustering analysis (see methods). This yields 258 clusters, 64 of which contained only a single gene. Of the 194 clusters with more than one gene, six (*P* [of observing as many or more clusters] = 0.34) and 104 (*P* = 0.01) have greater than 70% of the genes from either human or mouse, respectively. Moreover, we find 41 clusters only in one of the two species (5 human-specific (*P* = 0.03) and 36 mouse-specific (*P* = 0.09). Interestingly, these do not correspond to the classification to OR gene subfamilies. In fact, inspection of a full-length protein sequence phylogenetic tree of OR genes reveals that clusters of OR genes with highly similar binding sites in our analysis are not often monophyletic, and often are not even in close proximity with one another (as illustrated in Figure 2A, B). Specifically, only six of the 41 OR clusters specific to one species consist entirely of genes that form a monophyletic clade in the phylogenetic tree based on the entire protein sequences of human and mouse OR genes.

### Clustering analysis and the case of isovaleric acid

**Figure 3:**
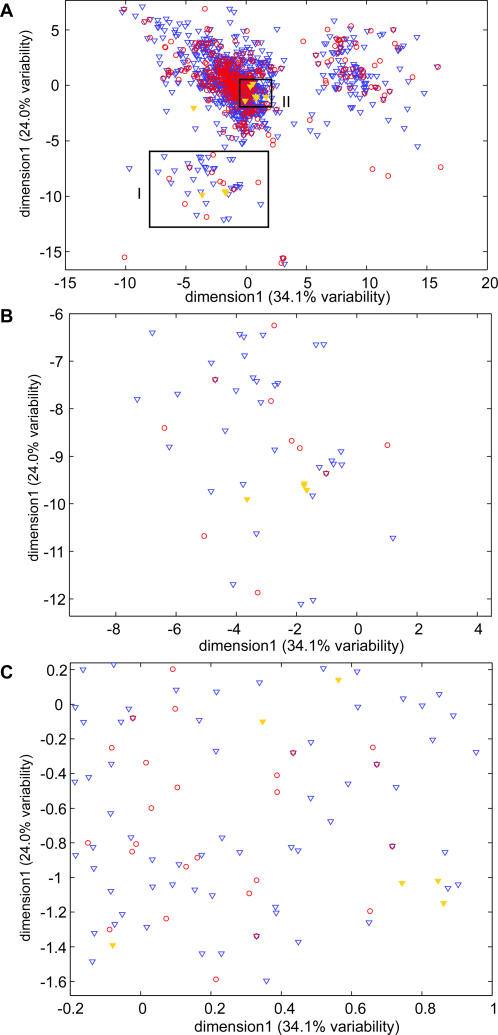
2D representation of the human (red) and mouse (blue) odorant spaces. The ‘Iva mouse OR genes’ (yellow) do not form a single cluster (A). High resolution 2D representations of map regions I (B) and II (C) reveal that human OR binding sites are interspersed with the mouse Iva ORs in both regions.

Our analysis suggests an explanation of the apparent contradiction between the sensitivity of mice and humans to isovaleric acid (IVA) and the lack of IVA OR orthologs in humans when full length sequences are compared. Specifically, the genomic regions responsible for specific anosmia to Iva in the mouse were identified [29] and, following the classification of the complete mouse OR gene repertoire, 12 intact OR genes (as well as two OR pseudogene with more than two coding region disruptions) were mapped to these regions [7]. Based on full-length protein sequence analysis, the 12 intact OR genes were classified into two small subfamilies that were found to be specific to mouse [7]. Based on the view that sub-family classification is a proxy for functional similarity, the lack of human OR gene orthologs to either of the two subfamilies would indicate that humans cannot detect Iva. However, O'Connell et al. [30] reported that humans can detect Iva at relatively low threshold concentration. Using the putative binding sites to cluster OR genes helps resolve this apparent contradiction: By our approach, the 12 mouse OR genes that confer the ability to detect Iva cannot be clustered without including human OR genes *i.e.,* genes from the two species are interspersed (Figure 3). Thus, consideration of only the binding sites suggests that genes with similar binding properties are present in both species and that both humans and mice can detect Iva (possibly with similar capacity), in agreement with the finding of O'Connell et al.

## Discussion

To date, the functional variability within and between OR gene repertoires has been assessed by considering overall protein similarity [7], [14]. However, following gene duplication, the binding properties of an OR gene (or any receptor) may be modified by a few changes to the binding site. As a result, full-length protein sequence phylogenetic analysis captures the evolutionary relationship between different OR genes, but not necessarily the similarity in binding properties. In other words, recently duplicated OR genes may be highly similar at the overall protein level, but differ markedly in their binding capacities due to few changes to their binding sites. Here, we assess functional variability within OR gene repertoires by comparing the putative binding sites [25] of all human and mouse putatively functional OR genes.

We find that the odorant spaces of the two species largely overlap, but that there is a somewhat larger than expected number of human-specific clusters of genes with highly similar binding sites, as well as a highly significant number of mouse-enriched (>70%) clusters. These clusters of genes do not form monophyletic, species-specific lineages when the entire OR protein sequences were analyzed. The finding that two sets of assumptions yield such disparate results is interesting in its own right as it calls into question the reliability of the standard subfamily-based classification of OR genes into functional groups.

It also raises the question of how to interpret OR gene clusters. A single receptor is thought to bind a series of chemically related odorants, with different affinities associated with each interaction [31], [32]. If so, then, even with fewer functional receptors, humans may have preferentially retained ORs with broad specificities (‘generalists’) in order to maintain the ability to detect most odorants [33]. Alternatively, humans may have kept sets of OR genes that differ subtly in their binding sites in order to maintain strong binding affinity for each and every odorant in a group of chemically related compounds that are important in human evolution. Our analysis may be able to generate hypotheses to distinguish between these two possibilities: when a species requires the ability to detect and discriminate between chemically related odorants at low concentrations, this pressure should give rise to a cluster of OR genes with only subtly different binding sites. Conversely, if a region of the odorant space is represented by only one (or very few) ORs, this might suggest that most of the related odorants in that space are detected with low affinity (i.e., only at high concentration).

Overall, our analysis suggests that the odorant spaces of human and mouse largely overlap suggesting that, at saturating concentrations, both species are able to detect most odorants. In this respect, our results are consistent with the behavioral findings of Laska et al. [21]. However, we also find dozens of clusters of similar binding sites that are exclusive to one species, or nearly so. We speculate that these clusters reflect differences between human and mouse with respect to threshold detections of odorants bound by the OR genes that these clusters contain.

This said, we cannot test our predictions, (or the predictions based on the commonly used analysis of the entire coding region). Mapping receptor-odorant interactions has proven to be a challenging task. Indeed, only a few receptor-odorant interactions have been reported [32], [34], [35], and a large number of them relate to a single gene, OR I7 from rat. While the rat I7-odorant interactions have been reported in independent publications using different assays [36], most of the other putative receptor-odorant interactions have not yet been replicated, and were found using only one assay, often with unclear implications for in-vivo receptor-odorant interaction. Moreover, some of the reports have been contradictory. Thus, existing data are neither comprehensive nor reliable enough to be used for validation of our predictions. Since neither our approach nor that of using the full coding sequence can be validated experimentally, it is unclear which yields more accurate results.

Moreover, our findings are based on the current prediction of the OR protein SDR, which may change when a validated OR protein structure is available, or when other residues that determine the binding specificity are recognized. Clearly, our results are dependent on the identity of the 22 residues we analyze. However, our analysis can be easily repeated for a different set of residues.

In conclusion, our approach can not replace empirical studies of receptor-odorant interactions, but can help to generate prediction that can be tested empirically. In particular, as demonstrated for the case of the IVA, hypotheses generated using our approach may be quite different from those generated under the widely used classification approach which is based on the analysis of the entire OR protein sequence.

## Methods

### Prediction of the specificity determining residues

We rely on previous prediction of 22 specificity determining residues (SDRs) in OR proteins [25]. The assumption underlying the prediction of these SDRs was that these residues would be more conserved among orthologs, which are assumed to have a similar binding spectrum, than among paralogs, which are assumed to have a divergent binding spectrum. The implementation of this concept relied solely on protein sequence information. The same concept has been previously utilized to predict SDRs in bacterial transcription factors [37] and eukaryotic and eubacterial protein kinases [38]. In both cases the predictions were consistent with solved structures as well as experimental results. For OR proteins, no X-ray crystallographic structure has been determined and the only report of a mutation affecting specificity [35] is now in question [39]. Thus, the only validation of the predicted SDRs utilized information available from homologous GPCRs: In a homology model based on a high-resolution X-ray crystallographic structure of rhodopsin [40], the 22 putative SDRs occupy the region corresponding to the retinal binding site. In addition, 21 of the 22 predicted SDRs correspond to amino acid positions that were previously associated with ligand-binding in at least one other GPCR [25].

### Multiple sequence alignment of OR proteins

We used the alignment of [25], which contains 1441 protein sequences (402 and 1039 sequences from human and mouse, respectively). The alignment was constructed from all human and mouse OR protein sequences that span the seven putative transmembrane regions, contain no ambiguous residues, and no more than two coding region disruptions (in the text, we refer to all these as ‘intact’ genes). The approach taken in aligning the sequences was hierarchical in nature, combining automatic alignment of closely related sequences and the merger of such small alignments into larger alignments, each containing all the sequences belonging to a specific OR family. These family alignments were manually edited in order to ensure the correct alignment of the transmembrane regions, as well as of other OR protein motifs (such as an N-glycosylation site). Also, positions containing a gap in more than half of the members of the family were edited out. Finally, the family-wise alignments were manually merged into a single one, containing all 1441 OR sequences [25].

### Phylogenetic analysis

A neighbor-joining tree was constructed from the most reliable positions in the OR multiple sequence alignment (positions containing fewer than 1% gaps), using ClustalX v1.83 [41]. Trees were drawn using TreeExplorer (K. Tamura; http://evolgen.biol.metro-u.ac.jp/TE/TE_man.html).

### Mapping of the odorant spaces of human and mouse

Currently, there are no solved structures of OR proteins. Here, we rely on a prediction of the putative binding site based on the premise that orthologous OR genes are likely to have similar binding sites while the binding sites of paralogous OR genes are likely to be different [25]. This theoretical prediction is in general agreement with the empirical results of Katada et al. [42]. We extracted the 22 residues corresponding to the putative odorant binding site from each OR sequence in the multiple alignment [25]. Nine OR genes (human genes: OR9G3P, OR11J2P, OR2AE1, OR56B4; mouse genes: MOR188-2, MOR126-1, MOR126-2, MOR176-3, MOR204-25P) were found to contain a gap within the putative binding site, and were excluded from subsequent analyses.

Next, we constructed a distance matrix comparing the putative binding sites of all possible pairs of OR genes. In modeling the distance between binding sites, we used the Grantham chemical difference matrix [26]. It has been shown that differences between disease alleles and wild-type alleles computed using the Grantham matrix are on average greater than those observed between putatively neutral polymorphic alleles, as well as inter-species differences [43]. This suggests that protein distances predicted by this matrix are functionally relevant.

We downloaded from the AAIndex database [44] the three amino acid scales used in the construction of the Grantham chemical difference matrix [26], namely aax1:GRAR740101 (Composition), aax1:GRAR740102 (Polarity), and aax1:GRAR740103 (Volume). We used these indices to translate the putative binding site of each OR protein into a 66-dimensional vector. We then computed all pairwise distances between the vectors, using the standardized Euclidean distance as the distance measure, i.e., weighting each coordinate by the inverse of its variance.

In order to to visualize the 66-dimensional space in two- and/or three-dimensions, we used the Isomap algorithm for geodesic dimensionality reduction [27]. We downloaded the Isomap Matlab package from http://isomap.stanford.edu/. We then applied the IsomapII function (in Matlab/Math Works Inc v7 (R14)) to the previously computed distance matrix, using all available points as landmarks, and requesting that ten dimensions be calculated. In order to select the appropriate ε value, the neighborhood size parameter, we applied the tuning methodology described in [28]. Briefly, in this methodology, the algorithm is run multiple times, varying only the neighborhood size parameter, and recording each time the values of two cost- functions: the fraction of variance in geodesic distance estimates not accounted for by the Euclidean embedding and the fraction of points not included in the largest connected component of the neighborhood graph, and thus not included in the Euclidean embedding. The optimal neighborhood size is then determined as a tradeoff between these two cost functions. We found ε = 14.5 to be the optimal value for both the three-dimensional (accounting for 68.9% of the variance) as well as for the two-dimensional (accounting for 58.1% of the variance) embeddings. We confirmed the validity of the projection method by calculating the correlation of the matrix of standardized Euclidean distances based on the 66 dimensions with the Euclidean distances based on projections to two (r^2^ = 0.75) or three (r^2^ = 0.81) dimensions.

### Comparison of the mapping of odorant space obtained using the SDRs with those obtained using random sets of OR protein positions

In order to repeat our analysis using random sets of 22 residues, we used the set of sequence alignments that include only residues with no gaps as a pool, excluding the 22 SDRs themselves. This screening step resulted in a pool of 154 residues (of 327 positions in the original alignment) to choose from. Out of this pool we randomly chose five sets of positions, each of size 22. We then repeated the mapping procedure, using these random sets of positions instead of the SDRs, and reproduced figure 1 for each random set of residues (Figure S1).

### Cluster analysis

We applied agglomerative hierarchical clustering with average linkage, as implemented in Matlab/Math Works Inc v7 (R14), to the three-dimensional coordinates obtained from the Isomap procedure, using Euclidean distances as a distance measure. To determine the appropriate number of clusters, we utilized the unrefined L-method [45]. For this purpose, the score for a specific number of clusters was the distance between the two nearest clusters (*i.e.* the clusters that would be merged if we were to decrease the number of clusters by one). By plotting scores against the number of clusters we determined the knee of the curve, and thus the number of optimal clusters, to be 110.

The 110 clusters obtained ranged in size from 1 (singleton clusters) to 323 ORs. Because of the wide range of cluster sizes, we decided to re-apply the hierarchical clustering. In order to determine which clusters would be divided further, we used the unrefined L-method [45] again, this time plotting cluster size against cluster rank, where the rank is determined by size. We found that the knee of the curve corresponded to a cluster of size 46, and, thus, re-applied the hierarchical clustering procedure to only the seven clusters containing at least 46 OR genes. Our final analysis yielded 258 clusters, ranging in size from 1 to 29 (mean size: 5.56).

To test the significance of observing clusters specific to one species or enriched (>70%) in one species, we took the following approach: For each group of clusters that are specific or enriched in one species, containing N clusters of sizes s_1_≥s_2_≥…≥s_N_, we randomly permuted the species-assignments 100,000 times, and counted the number of permutations fulfilling the following conditions (excluding singletons):

1. M, the number of clusters belonging to class X in the random permutation, fulfills N≤M.
2. Let t_1_≥t_2_≥…≥t_M_ be the sizes of the clusters belonging to class X in the random permutation, then: t_i_≥s_i_ for every 1≤i≤N

## Supporting Information

Figure S1 (0.05 MB DOC)
