# Supplementary material for: A Framework for Exploring Functional Variability in Olfactory Receptor Genes
Source: PLoS One. 2007 Aug 1;2(8):e682. doi: 10.1371/journal.pone.0000682 (PMC1925143; doi:10.1371/journal.pone.0000682)

Supplementary Figure 1.

Comparison of the 2D mapping of the OR repertoires of human and mouse obtained using the SDRs with those obtained using random sets of OR protein positions. In all panels mouse ORs are represented by triangles, whereas human ORs are represented by circles. **A.** 2D mapping of the OR repertoires using the SDRs. Two mouse-specific clusters (red and magenta, corresponding to regions a and d in Figure 1 in the paper), and one human-specific cluster (blue, corresponding to region c of Figure 1) are highlighted. **Panels B to F** display 2D mappings of the human and mouse OR repertoires obtained using randomly selected groups of OR protein positions (see Methods), with the same ORs highlighted as in panel A.

A.
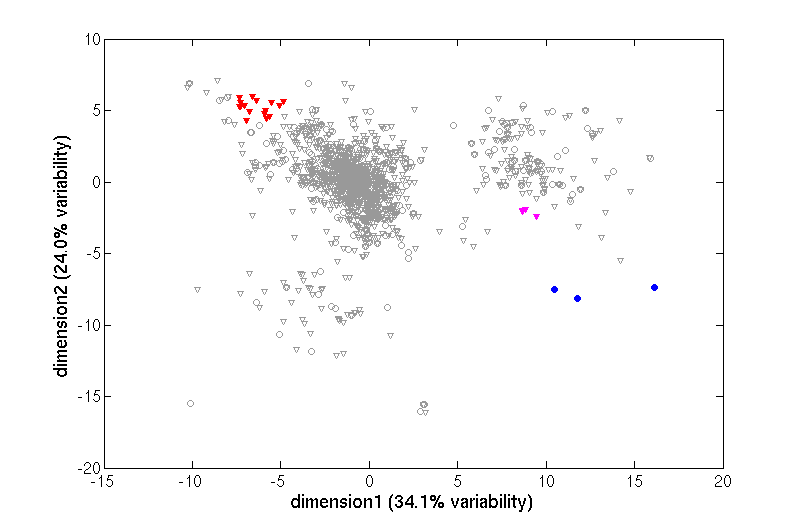


B.


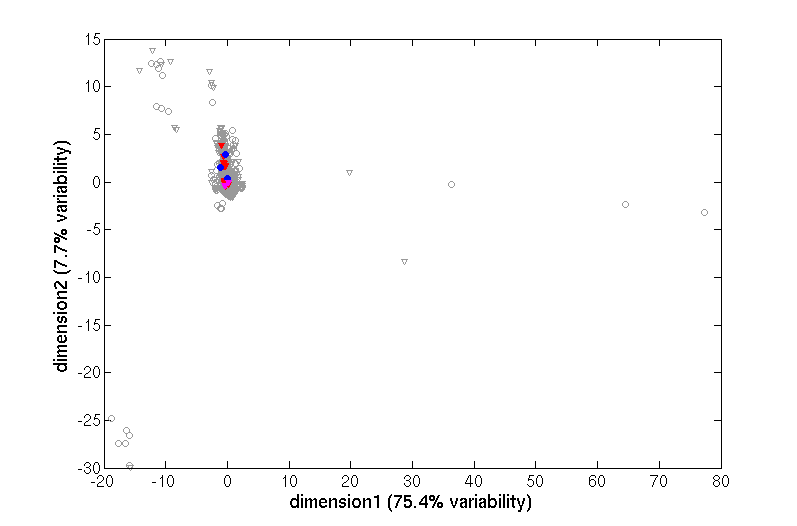


C.


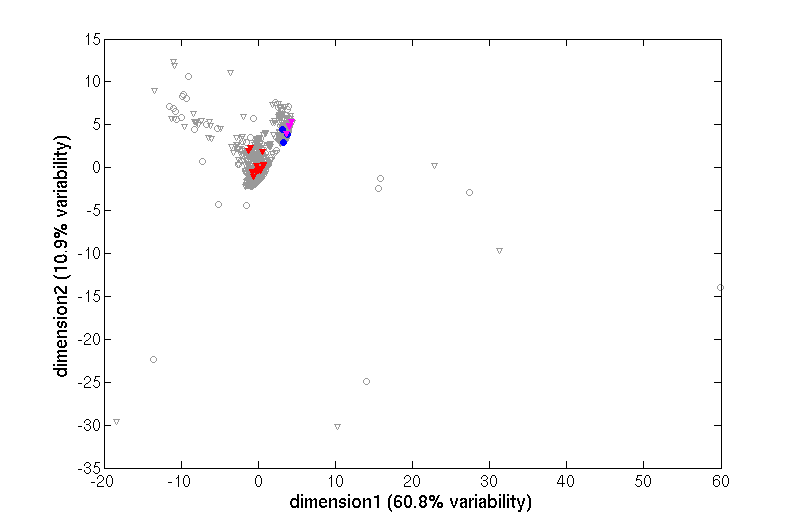


D.


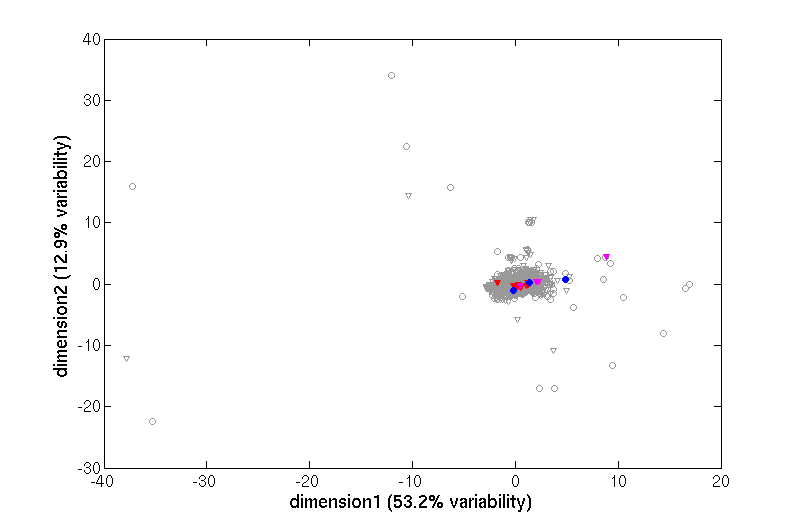


E.


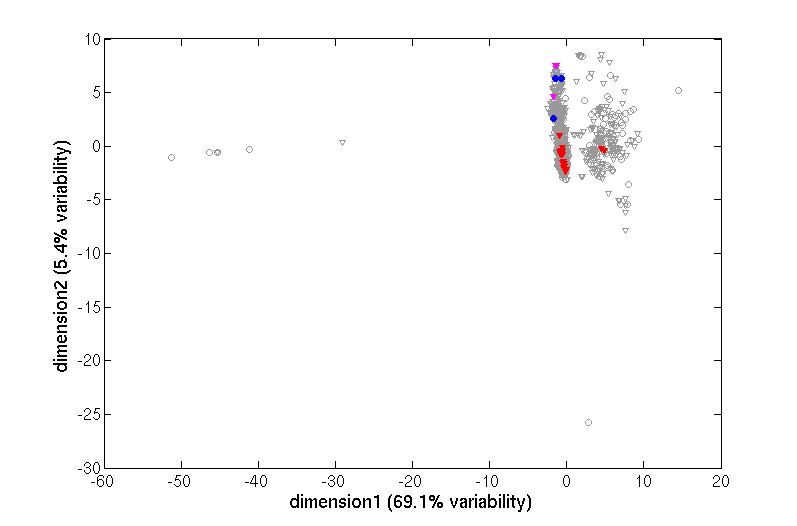


F.


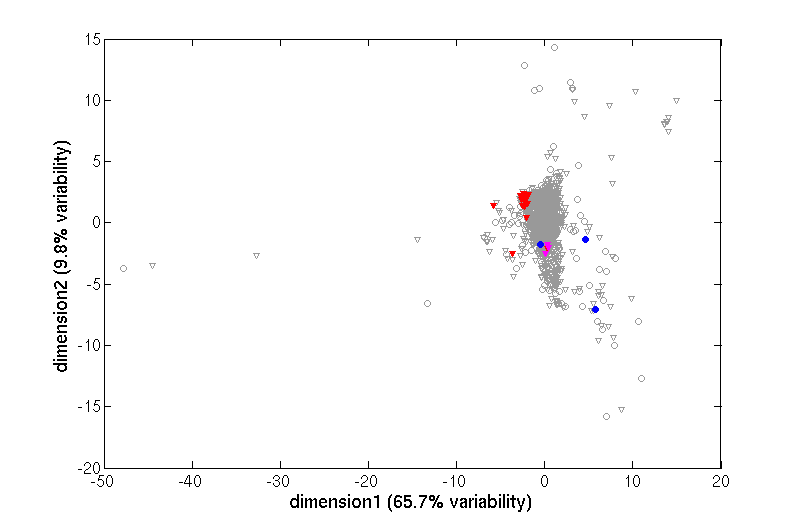

Supplement: Figure S1 — (0.05 MB DOC) [file pone.0000682.s001.doc]
